# Does immediate loading of a single implant in the healed anterior maxillary ridge improve the aesthetic outcome compared to conventional loading?

**DOI:** 10.1038/s41405-021-00083-4

**Published:** 2021-08-12

**Authors:** Radhika J Baireddy, Neil Cook, Siwei Li, Fadi Barrak

**Affiliations:** 1 School of Dentistry, University of Central Lancashire, Preston, England; 2 Visiting Specialist Services Academy Ltd, London, England

**Keywords:** Prosthetic dentistry, Dentistry

## Abstract

**Background:**

Immediate loading is an attractive option for avoiding secondary surgery. However, it is unclear whether it provides a better aesthetic outcome compared to conventional loading with implants placed in healed ridges.

**Aims:**

To compare the aesthetic outcomes of immediately and conventionally loaded single implants in healed anterior maxillary ridges.

**Methodology:**

A systematic review using PICO was conducted. EMBASE, MEDLINE and DoSS databases were searched. The Cochrane Risk of Bias tool for Randomised Controlled Trials and the Effective Public Health Practice Project tool for other study designs were used for quality appraisal. A narrative synthesis was undertaken.

**Results:**

A total of 622 articles were identified. After screening, a total of five papers were included. Results indicated no statistically significant difference in pink or white aesthetic scores between the immediate and conventional loading groups at 1- and 5-year review and the Papilla Index at the 1-year review.

**Conclusion:**

Within the limitations of this review, immediate loading of single implants provides a comparable aesthetic outcome to conventional loading in healed ridges of the anterior maxillary.

## Introduction

Missing single anterior teeth constitute >50% of referrals made to implant clinics.^1^ Patienst undergoing implant surgery have a strong desire to immediately restore function and aesthetics after implant placement with a restoration that resembles the adjacent teeth.^2–4^ Implants, when restored immediately after implant placement, can fulfil these desires.^2^ Immediate loading is when the restoration is placed at the time of surgery or within one week, thus bringing the implant into function.^5,6^ The immediate loading protocol is attractive to patients and clinicians due to potentially reduced treatment time, avoidance of second-stage surgery and the need to wear a removable provisional denture (RPD), thus bringing immediate comfort.^2,3,7^ Conventional loading is recommended for patients who require significant bone augmentation, have poor primary stability or are medically compromised.^8,9^ A systematic review of patients’ perspectives of implants placed immediately after extraction has demonstrated a 93% satisfaction rate pertaining to aesthetics for immediate loading and 91% for conventional loading.^10^

However, immediate implant placement may not be possible in some situations, such as where there is a large periapical lesion requiring significant bone healing or where there is a delayed presentation of the patient. In these circumstances, the implant is placed in healed ridges, two or more months following the removal of the tooth as opposed to immediately after extraction. To the authors’ best knowledge, there has not been a review of the aesthetic outcomes of immediate and conventional loading of implants placed in healed ridges.

This is relevant as conventional loading of implants placed in healed ridges requires a temporary prosthesis to replace the missing tooth during the healing period. A survey of patient priorities in implant treatment found that 30% of patients ranked avoidance of RPD after implant treatment as the top priority.^11^

A Cochrane review comparing immediate and conventional loading in implants placed in healed sites concluded no clinically significant difference in implant and prosthetic failure between the two groups.^6^ Other systematic reviews demonstrated comparable survival rates.^7,12^ The success and survival rates are comparable between immediate and conventional loading of implants placed in healed sites.^13^ The aim of this review is to compare the aesthetic outcome of immediate and conventional loaded single implants in healed ridges.

Clinical considerations for implant placement at different healing stages after extraction are unique and divergent.^7^ The healing of the socket is predictable up to 6–8 weeks; following that, bone healing occurs at a variable rate.^14^ Therefore, it will be beneficial if a comparison between immediate and conventional loading for healed sites in the maxillary aesthetic zone is made separately.^11^ The existing evidence comparing the aesthetic outcomes of immediate and conventional loading of dental implants is from implants placed at varied time points after extractions, which can introduce variables due to different healing stages.^6,12,15,16^ Two systematic reviews (Cheng et al.^12^ and Benic et al.^15^) have looked at implants placed in healed sites separately, comparing aesthetic outcomes between immediate and conventional loading. However, reported outcomes were from one Randomised Controlled Trial (RCT) only.^2^ Cheng et al.^12^ selectively reported on only one aesthetic outcome whereas the original RCT^2^ reported on three aesthetic outcomes. There have since been more RCT’s published on this topic.

Aesthetic outcomes of a single implant depend on the peri-implant hard and soft tissue.^12^ Pink Esthetic Index (PES), which assesses the peri-implant soft tissue;^17^ White Esthetic Index (WES), which is an aesthetic scoring of the implant crown;^18^ and Papilla Fill Index (PFI)/Papilla Index (PI)^19^, which assesses the size of the gingival papilla, are widely reported aesthetic indices in implant dentistry.^8^

Patients are more likely to be concerned about immediate loading in the upper aesthetic zone than the lower.^1^ The aesthetic outcome is dependent on the soft tissues and bone contour around the implant^17,18^ (the difference in the width of keratinised tissue around upper and lower teeth^20^ can cause different PES and WES scores between the two regions). Therefore, this study aimed to compare the aesthetic outcome of the immediately and conventionally loaded single implants in healed ridges in the anterior maxilla.

## Review question

PICO was used to frame the review question. Patient: adults undergoing rehabilitation of single edentulous space in the healed anterior maxillary ridge with implant-supported restoration; Intervention: Immediate implant-supported fixed provisional restoration; Comparator: Delayed implant-supported fixed provisional restoration and Outcome: Aesthetics outcome (including PES, WES and PI score).

Based on the existing literature, a hypothesis might be formulated that immediate loading of a single implant in the healed anterior maxillary ridge improves the aesthetic outcome when compared to conventional loading.

## Methods

### Design

Following the initial scoping searches and formulation of the research question, Dentistry and Oral Sciences Source (DOSS) and MEDLINE were searched using the EBSCOhost platform on 22/02/2020. EMBASE was searched using the Ovid interface on 27/02/2020. Table 1 presents the search strategy for MEDLINE. Search strategy for EMBASE and DOSS databases are presented in Supplementary Tables 1 and 2. The searches were supplemented with hand searching of the references in the existing systematic reviews.^6,7,12,15,16^ A PRISMA (Preferred Reporting Items for Systematic Reviews and Meta-Analyses)^21^ workflow was used.

**Table 1:** Search strategy for MEDLINE (HOST: EBSCO).

Immediate implant-supported fixed provisional restoration.	Immediate (temporary or provisional or interim) N5 (crown or restoration or prosthesis) (MH “dental prosthesis, implant-supported”) (MH “dental restoration, temporary”) Immediate loading
Aesthetic scores	“Pink? esthetic score*” PES “Modified pink? esthetic score” “Mod*PES” “White? esthetic score*” WES “Papilla index N3 score*” “?esthetic* N2 outcome”
Delayed implant-supported fixed provisional restoration.	Delayed (temporary or provisional or interim) N5 (crown or restoration or prosthesis) (MH “dental prosthesis, implant-supported”) (MH “dental restoration, temporary”) Delayed loading
Single implant	Implants* “single N3 implant” “endosseous implant*” (MH “dental implants”) (MH “dental implants, single-tooth”)
**#**	**Query**
S24	S5 AND S12 AND S17 AND S23
S23	S18 OR S19 OR S20 OR S21 OR S22
S22	(MH “dental implants, single-tooth”)
S21	(MH “dental implants”)
S20	TX “endosseous implant*”
S19	TX “single N3 implant”
S18	TX implants*
S17	S13 OR S14 OR S15 OR S16
S16	TX delayed loading
S15	(MH “dental restoration, temporary”)
S14	(MH “dental prosthesis, implant-supported”)
S13	TX Delayed (temporary or provisional or interim) N5 (crown or restoration or prosthesis)
S12	S6 OR S7 OR S8 OR S9 OR S10 OR S11
S11	TX WES
S10	TX “White? esthetic score*”
S9	TX “Mod*PES”
S8	TX “Modified pink? esthetic score”
S7	TX PES
S6	TX “Pink? esthetic score*”
S5	S1 OR S2 OR S3 OR S4
S4	Immediate loading
S3	(MH “dental restoration, temporary”)
S2	(MH “dental prosthesis, implant-supported”)
S1	TX immediate (temporary or provisional or interim) N5 (crown or restoration or prosthesis)

Asterisk (*) and question mark (?) were used as wildcards to maximise search results. An asterisk (*) was used to specify any number of characters at the end of a root word. A question mark (?) was used to represent a single character, anywhere in the word when there are variable spellings for a word.

### Inclusion criteria

- Adults (minimum 18 years old with no upper limit)
- Single implant-supported restoration in the maxillary aesthetic zone (UR5 to UL5)
- Partially edentulous patients
- Provision of implant-supported fixed provisional restoration
- Implants placed in healed sites
- Aesthetic outcome measured
- English language
- Randomised controlled trials
- Prospective cohort studies
- Quantitative studies.

### Exclusion criteria

- Case series/case studies
- Edentulous patients
- Implant-supported multi-unit bridge in the anterior region
- Removable provisional or direct, definitive restorations
- Studies with no aesthetic outcomes
- Implants placed in the mandible anterior region16
- Combined data from Type 1, Type 2, Type 3 and Type 4 placements
- Studies reporting separate bone augmentation procedure
- Combined data from mandibular and maxillary implants.

### Data extraction

Study characteristics including study design, participant characteristics, inclusion criteria, exclusion criteria, method of randomisation, type and timing of implant placed, site of implants, surgical procedure, soft tissue conditioning with a temporary crown, the timing of definitive crown in immediate loading group, the timing of definitive crown in delayed loading group, aesthetic outcome measures and loss to follow-up/excluded were extracted. The full data extraction table is presented in Supplementary Table 3. The mean PES, mean WES and percentage of implants with complete papilla at all available review time points were extracted for the immediate and delayed loading groups.

### Analysis/synthesis

Cochrane Risk of Bias Tool for Randomised Controlled Studies was used to appraise Randomised Control Trials.^22^ Effective Public Health Practice Project (EPHPP) Quality assessment tool for quantitative studies^22^ was used to appraise cohort studies. A descriptive synthesis was undertaken due to the heterogeneity of the included studies. Subgroup narrative analysis was undertaken based on the scoring systems (PES, WES and PI).

## Results

A total of 623 studies were identified. Fifteen were duplicates, so abstracts and titles of 608 papers were assessed. Five hundred and ninety studies were excluded at this stage. Full-text articles of 18 studies were obtained and analysed for inclusion in the review. Based on the exclusion criteria, six RCT’s were excluded as the implants were directly loaded with definitive restoration rather than provisional restorations.^23–28^ Two studies had from both maxillary and mandibular teeth.^29,30^ Two studies did not report the aesthetic outcome.^31,32^ Two studies looked at early implant placement.^18,33^ One was a case report.^34^ A table of excluded studies with detailed reasoning and references is presented in Supplementary Table 4.

**Fig. 1:**
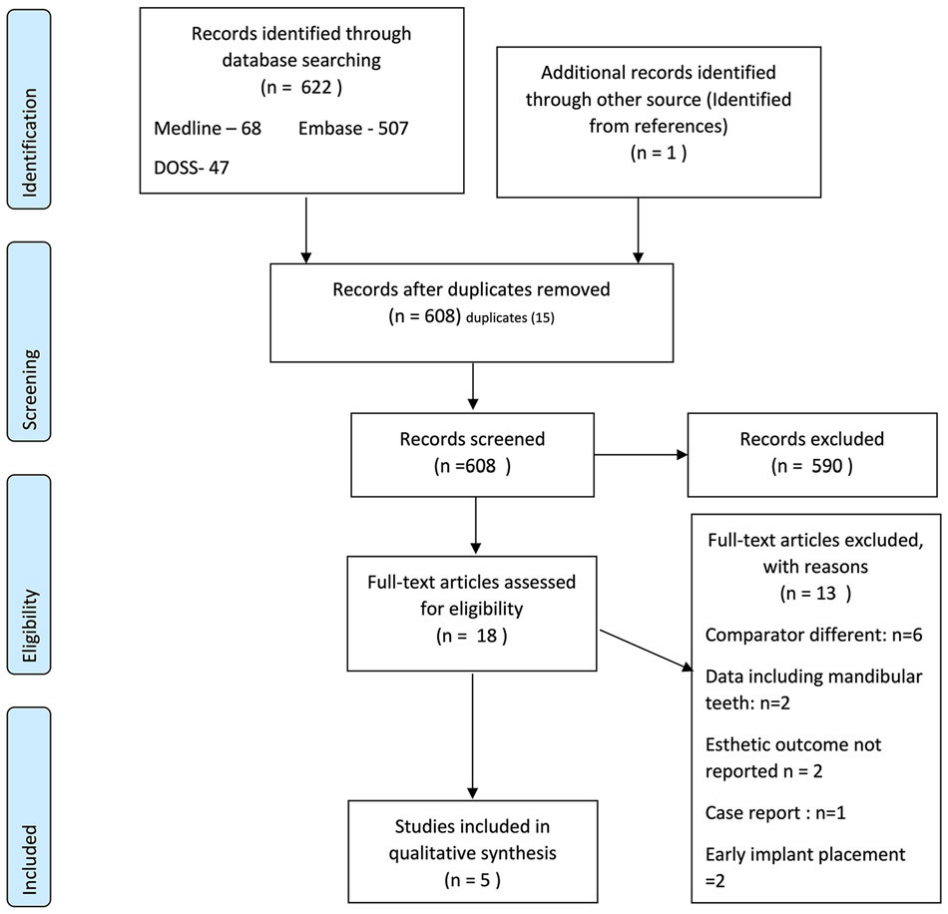
PRISMA fowchart for selecting eligible studies. A total of 5 studies were enrolled in this systematic review.

Following the PRISMA^21^ workflow chart (Fig. 1), five studies were included in this review after assessing the inclusion and exclusion criteria.^2,35–38^

Study characteristics are presented in Table 2. Three studies were RCTs, and two were prospective cohort studies. The sample size varied between 23 and 94. Mean age was similar across all studies, being around 40 years. Single implants were placed in the maxilla from the upper right second premolar to left second premolar in healed ridges. All the implants were restored with a temporary crown (immediate or delayed) followed by a definitive crown. All the studies assessed aesthetic outcomes. The summary of the findings is shown in Table 3. Tables 4 and 5 illustrate the risk of bias.

**Table 2:** Study characteristics.

	Gjelvold et al. 2017	Den Hartog et al. 2016	Heydecke et al. 2019	Hall et al. 2007	Raes et al. 2018
Study design	RCT - parallel groups	RCT- parallel groups	Prospective cohort study	RCT - parallel groups	Prospective cohort study
Number of participants	50	62	94	28	23
Setting	Centre of Dental specialist care, Malmo	Oral and Maxillofacial surgery of the university medical centre, Groningen	Eight participating centres (Austria, Germany, Italy, Serbia and The USA)	Oral Implantology Area of research strength, University of Otago, New Zealand.	University Hospital, Ghent
Participant allocation	Parallel design 25—Immediate load 25—Delayed load	Parallel design 31—Immediate load 31—Delayed load	Prospective single cohort 99 implants- placed in 94 patients (5 patients received 2 implants each)	Parallel design 14—Conventional restoration group 14—Immediate restoration group	Prospective cohort IIT—Immediate Implant treatment—16 CIT—Conventional implant treatment—23
Participants age Mean ± years (range)	Immediate group Mean age 40.8 ± 13.3 (19.0–66.6) Delayed group Mean age 40.9 ± 15.5 (18.5–76.7)	Immediate load Mean age 38.4 ± 14.0 (18–66) Delayed load Mean age 40.1 ± 14.4 (18–67)	Mean age 41.1 ± 14.3 years (range 18–79 years)	Mean age 43.3 years (range 21–71 years) The paper mentions “No statistical difference between both groups for age”	IIT—mean age 45; age range 22–68 CIT—mean age 40; age range 19–75
Participants sex	Immediate group 14 Men/11 Women Delayed group 6 Men/19 women	Not mentioned	57.4% female and 42.6% male	The paper mentions “No statistical difference between both groups for gender”	IIT—10 men, 6 women CIT—12 men, 11 women
Site of implants	Incisor, Canine or premolar in Maxilla Paper does not mention the number for each site	Central incisor- 14 Lateral incisor- 10 Canine- 4 First Premolar- 3	Central incisor 9 Lateral incisor- 17 Canine- 7 First Premolar- 33 Second premolar- 33.	Incisor, Canine, first premolar or second premolar in Maxilla Paper does not mention the number for each site	Central incisor- 3 IIT, 3 CIT Lateral incisor- 5IIT, 10 CIT Canine- 2 IIT, 1 CIT First premolar- 6 IIT, 9 CIT
Bone condition	Healed bone (4 or more months after extraction)	Healed bone (3 or more months after extraction)	Healed bone (≥2 months after extraction)	Healed bone	Healed bone (>3 months after extraction)
Type of implant placed	Tapered internal implants (BioHorizons)	Nobel replace tapered groovy implant (Nobel Biocare)	Nobel Biocare	Southern implants- tapered implants (2.5–4 mm) Roughened surface Sa of 1.43 µm	Astra tech implant system, Osseospeed.
Intervention: type and timing of immediate temporary crown	Screw retained – Titanium temporary abutment with a composite crown *Immediate*	Screw retained temporary crown. *Within 24* *h of implant placement*	A cement- or screw-retained provisional 86 implants- temporary abutment with temporary crown were placed immediately. 13- final abutments with temporary crowns were placed immediately. *Within 24* *h following surgery*	Screw retained provisional crown. *Within 4* *h of Implant surgery*	Titanium direct abutment and acrylic crown Cement retained provisional crown with temporary cement (temp bond) *Immediate*
Occlusion of the immediate temporary crown	Light centric contact relation and free from eccentric contacts with opposing teeth	Crown was free from centric contacts with opposing teeth	Functionally loading	Free of contacts in maximum intercuspation and excursions	Clear of all contacts in centric occlusion and eccentric movements.
Comparator: type and timing of delayed temporary crown	Screw Retained – Titanium temporary abutment with a composite crown *4 months*	Screw retained temporary crown. *3 months of healing*	Not applicable	Screw retained provisional crown. *26 weeks of healing*	Not applicable
Timing of definitive crown in immediate loading group	2 months from implant installation	6 months from implant installation	Within 6 months after implant placement	8 weeks pf provisionalization	10 weeks following implant surgery
Timing of definitive crown in delayed loading group	After 8 months of implant placement	6 months from implant installation	Not applicable	8 weeks pf provisionalization	Not applicable
Definitive crown	Customised Zirconia abutment with titanium base, cemented or screw retained	Customised Zirconia abutment cemented, or screw retained	Cement or screw retained NobelProcera crown with a titanium or zirconia abutment	Screw retained metal ceramic crown on hexed old cylinders	Titanium abutment and full ceramic crown Cement retained crown
Outcome assessed.	Primary (PES, WES and Papilla Index)	Secondary (PES, WES, and Papilla Index)	Secondary outcome (Papilla Index and PES)	Primary (Papilla Index)	Primary (PES and WES)

**Table 3:** Summary of findings.

Outcome	Gjelvold et al. 2017 Number of participants: 50 Immediate Delayed		Den Hartog et al. 2016 Number of participants: 62 Immediate Delayed						Heydecke et al.2019 Number of participants: 94 Immediate	Hall et al. 2007 Number of participants: 28 Immediate Delayed		Raes et al. 2018 Number of participants: 23 Immediate	Overall quality
PESAt 1 year Mean ± SD (Range)	10.36 ± 2.46 (Range 3–14)	10.67 ± 2.32 (Range 5–14)	7.1 ± 1.5 (Range 3–10)				6.5 ± 1.6 (Range 4–9)		9.69 ± 2.04	Not an outcome measure		9.70 ± 1.72	Moderate
	*P* = 0.700		*P* > 0.05										
At 2 years Mean ± SD	Not followed		Not a review appointment						10.04 ± 1.98				
At 3 years Mean ± SD	Not followed		Not a review appointment						9.87 ± 2.1				
At 5 years Mean ± SD Range	Not followed		7.2 ± 1.5 (Range 3–10)					6.8 ± 1.3 (Range 5–9)	Not yet reviewed				
			*P* > 0.05										
At 8 years Mean ± SD	Not followed		Not a review appointment						Not a review appointment			9.22 ± 2.31 *P* = 0.763 bet 1–8 years	
WESAt 1-year Mean ± SD (Range)	7.76 ± 1.30 (Range 5–10)	7.87 ± 1.39 (Range 5–10)	7.8 ± 1.5 (Range 4–10)		7.6 ± 1.6 (Range 4–10)				Not an outcome measure	Not an outcome measure Not an outcome measure		7.00 ± 2.37 (Range 2–10)	Moderate
	*P* = 0.724		*P* > 0.05										
At 2 years Mean ± SD	Not followed		Not a review appointment										
At 3 years Mean ± SD	Not followed		Not a review appointment										
At 5 years Mean ± SD Range	Not followed		7.9 ± 1.2 (Range 5–9)	7.7 ± 1.2 (Range 5–10)									
			*P* > 0.05										
Papilla index Percentage of complete papilla fill at 1 year	28%	46%	43.3%		33.9%				90.6%	Not given	Not given	Not an outcome measure	Moderate
	*P* = 0.244		The study states “No statistical difference” no data provided							The study states “No statistical difference” no data provided			
Percentage of complete papilla fill at 2 year	Not followed		Not a review appointment						92.8%	Not followed			
Percentage of complete papilla fill at 3 year	Not followed		Not a review appointment						88.6%	Not followed			
Percentage of complete papilla fill at 5 year	Not followed		36.5%			25.9%			Not a review appointment	Not followed

**Table 4:** Risk of bias for Gjelvold et al. 2017, Hall et al. 2007 and den Hartog et al. 2016.

	Random sequence generation.	Allocation concealment	Blinding of participants and personnel	Blinding of outcome assessment	Incomplete outcome data	Selective reporting	Other sources of bias
Den Hartog et al. 2016							
Gjelvold et al. 2017							
Hall et al. 2007							

LOW RISK  UNCLEAR RISK  HIGH RISK .

**Table 5:** Risk of bias for Heydeck et al. 2019 and Raes et al. 2018.

Components of EPHPP	Heydecke et al. 2019	Raes et al. 2018
Selection bias	MODERATE	MODERATE
Study design	MODERATE	MODERATE
Confounders	MODERATE	MODERATE
Blinding	WEAK	MODERATE
Data collection methods	STRONG	STRONG
Withdrawals and drop-outs	WEAK	MODERATE
Global rating for this paper	WEAK	STRONG

### Pink esthetic score (PES)

All the RCT’s that compared immediate loading with conventional loading identified no clinically significant statistical difference in the PES at 1 year (den Hartog et al.^2^, Gjelvold et al.^35^) and at 5 years (den Hartog et al.^2^). Cohort studies reported comparable PES for the immediately loaded implants to the contralateral tooth (Heydecke et al.^37^ Raes et al.^38^). All studies except one study (Hall et al.^36^) reported PES as an aesthetic outcome.

A statistically significant improvement in PES from the baseline score (definitive crown fit stage) to the 1-year review was noted in both the groups (den Hartog et al.^2^, Gjelvold et al.^35^). Heydecke et al.^37^ and Raes et al.^38^ reported an improvement from baseline in the immediately loaded cohort at 1-year review, and PES stabilised for the next two years; these studies did not report conventional loading. Den Hartog et al.^2^ reported an improvement in the mean PES from 1-year review to 5-year review in both groups.

Raes et al.^38^ reported no statistical difference in the PES between the 1-year review and 8-year review for the immediately loaded cohort; the study did not report outcome for the conventional loading cohort. Raes et al.^38^ and Gjelvold et al.^35^ used the original indexing method (PES) by Furhauser et al. Den Hartog et al.^2^ used the modified version of PES by Belser et al.^18^ Heydecke et al.^37^ failed to mention the type of PES indexing used.

### White esthetic score (WES)

Only three studies reported WES as an outcome measure (den Hartog et al.^2^, Gjelvold et al.^35^, Raes et al.^38^). The RCT’s comparing immediate and conventional loading reported no statistically significant difference for WES between the groups at the 1 year (den Hartog et al.^2^, Gjelvold et al.^35^) and at 5 years (den Hartog et al.^2^) review appointments. Raes et al.^38^ reported 40% of the immediately loaded implants showed almost perfect WES score, 40% showed acceptable WES and 20% unfavourable WES scores at the 1-year review. Raes et al.^38^ did not report 8-year review data for WES, and the study did not report conventional loading.

Raes et al.^39^ reported a high percentage (20%) of aesthetic failures WES ≤ 5; the mismatch of the colour of the crown was the most common reason for this failure.

### Papilla index (PI)

All the RCT’s considered PI as an aesthetic outcome and reported no statistically significant difference between immediate and conventional loading at 1-year review appointment (den Hartog et al.^2^, Gjelvold et al.^35^, Hall et al.^36^). The statistical data for comparison between the immediate and conventional group at 5 years review is not available (den Hartog et al.^2^). The cohort study reporting PI scores for the immediately loaded group reported 90.6% of the implants had complete papilla fill at 1 year (Heydecke et al.^37^). Raes et al.^39^ did not consider PI as an aesthetic outcome.

Papillary Index had the most heterogeneous data reported. An RCT conducted by Hall et al.^36^ used a modified papilla index scoring. The study did not report individual scoring for the immediate and conventional group, and it reported combined papilla indices either remained unchanged (28%) or improved (63%) at 1- year review. Den Hartog et al.^2^ reported that 43.3% of implants had a complete papillary fill in the immediate group compared to 33.9% in the conventional group at the 1-year review. At 5 years, the percentage was further reduced to 36.5% (immediate) and 25.9% (conventional). On the contrary, the multicentred cohort study reported high scores of 90.6% at 1 year, 92.8% at 2 years and 88.6% at 3 years (Heydecke et al.^37^)

The included studies were of low-medium risk of bias in the sequence generation and allocation concealment.^2,35–38^ Only den Hartog et al.^2^ and Raes et al.^38^ blinded the outcome assessor. The studies have low to high risk for incomplete outcome reporting, and selective reporting.^2,35–38^ Risk of bias tables are presented in Tables 4 and 5. The studies had limitations. The study by Heydecke et al.^37^ did not mention the scoring system used for PES. Two types of indexing methods with different scoring criteria are described in the literature for PES.^17,18^ The studies by Gjelvold et al.^35^ and Raes et al.^38^ used PES scoring system by Furhauser et al.^17^ while den Hartog et al.^2^ used the PES scoring system by Belser et al.^18^ PES scoring system described by Furhauseret al.^17^ asses the peri-implant soft tissue at seven variables; each variable is scored between 0 and 2 with a maximum score of 12. Belser et al.^18^ asses the peri-implant soft tissue at 5 variables; each variable is scored between 0 and 2 with a maximum score of 10. Gjelvold et al.^35^ and Hall et al.^36^ reported only 1-year review data. Hall et al.^36^ presented combined data of both immediate and conventional loaded groups making it difficult to compare data. Raes et al.^38^ selectively reported data; only PES was reported in the 8 years review paper. 1-year results of the same prospective cohort study were published in another paper, and it reports both PES and WES data.^39^ No statistically significant difference was found between 1 year and 8 years follow up for PES (*p* ≥ 0.470). The clinical protocol followed across the studies also varied considerably, and due to the heterogeneous nature of the data, a meta-analysis was not possible.

## Discussion

The aim of this review was to compare the aesthetic outcome of immediate and conventional loaded single implants in healed ridges. The hypothesis that immediate loading of a single implant in the healed anterior maxillary ridge improves the aesthetic outcome when compared to conventional loading was rejected. As a body of evidence, when PES, WES and PI were all considered, the included studies in this review suggest that immediate loading of single implants placed in healed sites of the maxillary aesthetic zone provides comparable aesthetics to conventional loading of implants in the short term (up to 5 years).

The finding is in contrast to some suggestions that immediately loaded implants provide better aesthetics as the healing of the soft tissues occurs against the natural shape of the provisional restoration.^3^ One possibility could be that the implants were placed in healed sites.^13^ Chappuis et al.^39^ described the dimensional changes in the facial bone after extraction. The extent of resorption affects the soft tissue anatomy at the implant site.^40^ The amount and appearance of the soft tissue before implant placement might be more relevant for aesthetics than the timing of loading.^2^

Primary stability is an essential factor before considering the immediate loading of an implant.^14^ Conventional loading should be considered if primary stability is not achieved or in the presence of poor prognostic factors.^3^ The authors of this review acknowledge that in addition to the timing of loading, the aesthetic outcome of a single implant-retained restoration is dependent on multiple factors. The surgical skill of the surgeon is a confounding factor for achieving a high standard aesthetic outcome.^41^ The study by Heydecke et al.^37^ was carried out at multiple centres by surgeons with a varied skill set; this is considered as a limitation by the study. Hall et al.^36^ had both experienced and trainee surgeons who performed the implant surgery. However, it does not report the individual’s scores for each surgeon. These could lead to heterogeneity due to different levels of surgical skills of the operators. According to Busser et al.^41^, aesthetic failures can be minimised by proper patient selection and training of the surgeon. An ideal 3-dimensional prosthetic driven implant position is essential to achieve good soft tissue stability and aesthetics.^3^ Clinicians should ensure ≥1.5 mm of buccal bone to maintain aesthetics over the long term.^42^ Raes et al.^38^ noticed a reduction of buccal bone after eight years of implant placement in the immediately loaded cohort; however, the PES index remained stable/comparable to conventional loading.

Flap design and soft tissue augmentation are other confounding factors for aesthetics. Esposito et al.^43^ suggested there is no significant difference between types of flap designs for aesthetics and that sites receiving soft tissue grafting achieved better aesthetics. Lin et al.^20^ showed that lack of sufficient keratinised tissue around an implant is associated with tissue inflammation, mucosal recession and attachment loss, which in turn affects the aesthetics. Amongst all immediately loaded cohorts, authors observed Heydecke et al.^37^ reported a higher percentage of implants with complete PI scores compared to the other studies included in this review. It is not clear if this can be attributed to different flap designs or soft tissue augmentation used in the study by Heydecke et al.^37^ Another factor is guided bone regeneration. A recent study on beagle dogs has found a minimum thickness of 1.5 mm of buccal bone is required to prevent physiological and pathologic bone loss.^42^ Four of the studies in this review have performed guided bone regeneration during the implant placement surgery to improve the thickness of the buccal bone^2,35–38^ for both immediate and conventional loading. Guided bone regeneration might contribute to a good aesthetic outcome reported for both immediate and conventionally loaded implants. Soft tissue conditioning and emergence profile using the provisional restoration is another factor contributing to aesthetics. The provisional crown is used to shape the soft tissues around the implants so that the final restoration has an ideal emergence profile.^44,45^ The evidence is sparse on this topic.^46^ Only one study by Gjelvold et al.^35^ carried out soft tissue conditioning using provisional crown for the conventional group. As the studies reported in this review are heterogeneous, the effect of confounding factors on the aesthetic outcomes should be considered. Despite the heterogeneity of confounding factors found in the studies reported in this review, the findings from the PES, WES or PI indices suggest immediate loading provide comparable aesthetic outcomes to conventional loading.

### Strengths and limitations of the review

Unlike previous reviews in this area which have included mixed data maxillary, mandibular teeth^6,12,15,16^ and all types of placement protocols, this review presents results related to a specific clinical situation (implants placed in healed ridges of the aesthetic zone of the maxilla). The previous systematic reviews^12,15^ on this topic have drawn conclusions based on only one RCT, whereas this systematic review has collected data from three RCT’s and two prospective cohort studies looking at the same clinical situation. A dental specific database, DOSS, was also used to identify papers.

The review only included papers in the English language; this could be a limitation as important information published in other languages could be missed. The review looked at implants placed in healed sites only, so the results would not apply to immediate (Type 1) and early (Type 2) placement protocols. The overall quality of evidence for each outcome is moderate based on critical analysis. The evidence included three RCT’s and two prospective cohort studies. All studies included in this review had a similar design, in which implant placement was followed by either immediate or conventional provisional restoration and then definitive restoration. Based on the findings in this review, the authors propose that future RCTs with strict inclusion criteria of the specific clinical situation with a large number of participants and long-term results will likely provide significant evidence on the aesthetic outcomes. A systematic review of aesthetic outcomes along with an outcome based on patient opinion (e.g. questionnaires) can potentially provide further insights. The reviewer would be able to compare PES, WES and PI indices with patients satisfaction, thus strengthening the decision-making process for the clinicians.

## Conclusion

Within the limitations of this systematic review, it can be concluded that immediate loading of the single implants placed in healed sites of the maxillary aesthetic zone provides comparable aesthetics to conventional loading of implants in the short term. Clinicians, however, should take caution while considering the findings of this review due to the noticeable heterogeneity in confounding factors of aesthetic outcome in the included studies.

### Benefits of the findings of the paper

1. The existing evidence comparing the aesthetic outcomes of immediate and conventional loading of dental implants is from implants placed at varied time points after extractions, which can introduce variables due to different healing stages. This paper looked at implants placed in healed sites as a separate entity, comparing aesthetic outcomes between immediate and conventional loading. The findings are based on five primary research papers on implants placed in healed sites.
2. The evidence from this review suggests that immediate loading of the single implants placed in healed sites of the maxillary aesthetic zone provides comparable aesthetics to conventional loading of implants in the short term. The findings of this review would help the clinician in the evidence-based decision-making process while choosing between immediate and conventional loading for single implants placed in healed sites.
3. PES, WES and PI indices of the aesthetic outcome were evaluated, and there was no clinically significant difference between the two groups.

## Supplementary information

Supplementary tables
