# Supplementary material for: Does immediate loading of a single implant in the healed anterior maxillary ridge improve the aesthetic outcome compared to conventional loading?
Source: BDJ Open. 2021 Aug 12;7:30. doi: 10.1038/s41405-021-00083-4 (PMC8360987; doi:10.1038/s41405-021-00083-4)
Supplement: Supplementary file 1 — Supplementary tables [file 41405_2021_83_MOESM1_ESM.pdf]

Supplementary Table 1. **Search Strategy for DoSS (HOST: EBSCO).**

**Immediate Implant supported fixed provisional restoration.**	Immediate (Temporary Or provisional or interim) N5(Crown or restoration or prosthesis)<br>DE "TEMPORARY restoration (Dentistry)"
**Aesthetic scores**	“Pink? esthetic score*”<br>PES<br>“Modified pink? esthetic score”<br>“Mod*PES”<br>“White? esthetic score*”<br>WES<br>“Papilla index N3 scores”<br>“? esthetic* N2 outcome”<br>“Midfacial mucosal level*”
**Delayed Implant supported fixed provisional restoration.**	Delayed (Temporary Or provisional or interim) N5(Crown or restoration or prosthesis)<br>DE "TEMPORARY restoration (Dentistry)"
**Single Implant**	Implants*<br>“Single N3 Implant”<br>“endosseous Implant*”<br>DE "DENTAL implants"

#	**Query**
S18	S3 AND S11 AND S12 AND S17
S17	S13 OR S14 OR S15 OR S16
S16	DE "DENTAL implants"
S15	TX “endosseous Implant*”
S14	TX “Single N3 Implant”
S13	TX Implants*
S12	TX Delayed (Temporary Or provisional or interim) N5(Crown or restoration or prosthesis)

S11	S4 OR S5 OR S6 OR S7 OR S8 OR S9 OR S10
S10	TX “Midfacial mucosal level*”
S9	TX WES
S8	TX “White? esthetic score*”
S7	TX “Mod*PES”
S6	TX “Modified pink? esthetic score”
S5	TX PES
S4	TX “Pink? esthetic score*”
S3	TX S1 OR S2
S2	TX DE "TEMPORARY restoration (Dentistry)"
S1	TX Immediate (Temporary Or provisional or interim) N5(Crown or restoration or prosthesis)

Supplementary Table 2. **Search Strategy for Embase (Ovid platform).**

**Immediate Implant supported fixed provisional restoration.**	Immediate provisional adj5 (Crown or restoration or prosthesis)<br>Immediate temporary adj5 (Crown or restoration or prosthesis)<br>Immediate interim adj5 (Crown or restoration or prosthesis)<br><br>tooth prosthesis/ or exp "dental prosthesis and implant"<br>Immediate loading
**Aesthetic scores**	Pink? esthetic score$<br>PES<br>Modified pink? esthetic score<br>Mod$ PES<br>White? esthetic score$<br>WES<br>Papilla index adj3 scores<br>? esthetic$ adj2 outcome<br><br>exp esthetics/
**Delayed Implant supported fixed provisional restoration.**	Delayed provisional adj5 (Crown or restoration or prosthesis)<br>Delayed temporary adj5 (Crown or restoration or prosthesis)<br>Delayed interim adj5 (Crown or restoration or prosthesis)<br><br>tooth prosthesis/ or exp "dental prosthesis and implant"<br>Delayed Loading
**Single Implant**	Implants$<br>Single adj3 Implant<br>Endosseous Implant$<br>exp implant/<br>exp single tooth implant/<br>exp endosseous implant/

30	6 and 16 and 22 and 29
29	23 or 24 or 25 or 26 or 27 or 28
28	exp endosseous implant/
27	exp single tooth implant/
26	exp implant/
25	Endosseous Implant$.mp. [mp=title, abstract, heading word, drug trade name, original title, device manufacturer, drug manufacturer, device trade name, keyword, floating subheading word, candidate term word]
24	(Single adj3 Implant).mp. [mp=title, abstract, heading word, drug trade name, original title, device manufacturer, drug manufacturer, device trade name, keyword, floating subheading word, candidate term word]
23	Implants$.mp. [mp=title, abstract, heading word, drug trade name, original title, device manufacturer, drug manufacturer, device trade name, keyword, floating subheading word, candidate term word]
22	17 or 18 or 19 or 20 or 21

21	Delayed Loading.mp. [mp=title, abstract, heading word, drug trade name, original title, device manufacturer, drug manufacturer, device trade name, keyword, floating subheading word, candidate term word]
20	tooth prosthesis/ or exp "dental prosthesis and implant"/
19	(Delayed interim adj5 (Crown or restoration or prosthesis)).mp. [mp=title, abstract, heading word, drug trade name, original title, device manufacturer, drug manufacturer, device trade name, keyword, floating subheading word, candidate term word]
18	(Delayed temporary adj5 (Crown or restoration or prosthesis)).mp. [mp=title, abstract, heading word, drug trade name, original title, device manufacturer, drug manufacturer, device trade name, keyword, floating subheading word, candidate term word]
17	(Delayed provisional adj5 (Crown or restoration or prosthesis)).mp. [mp=title, abstract, heading word, drug trade name, original title, device manufacturer, drug manufacturer, device trade name, keyword, floating subheading word, candidate term word]
16	7 or 8 or 9 or 10 or 11 or 12 or 13 or 14 or 15
15	exp esthetics/
14	(?esthetic$ adj2 outcome).mp. [mp=title, abstract, heading word, drug trade name, original title, device manufacturer, drug manufacturer, device trade name, keyword, floating subheading word, candidate term word]
13	(Papilla index adj3 scores).mp. [mp=title, abstract, heading word, drug trade name, original title, device manufacturer, drug manufacturer, device trade name, keyword, floating subheading word, candidate term word]
12	WES.mp. [mp=title, abstract, heading word, drug trade name, original title, device manufacturer, drug manufacturer, device trade name, keyword, floating subheading word, candidate term word]
11	White? esthetic score$.mp. [mp=title, abstract, heading word, drug trade name, original title, device manufacturer, drug manufacturer, device trade name, keyword, floating subheading word, candidate term word]
10	Mod$ PES.mp. [mp=title, abstract, heading word, drug trade name, original title, device manufacturer, drug manufacturer, device trade name, keyword, floating subheading word, candidate term word]
9	Modified pink? esthetic score.mp. [mp=title, abstract, heading word, drug trade name, original title, device manufacturer, drug manufacturer, device trade name, keyword, floating subheading word, candidate term word]
8	PES.mp. [mp=title, abstract, heading word, drug trade name, original title, device manufacturer, drug manufacturer, device trade name, keyword, floating subheading word, candidate term word]
7	Pink? esthetic score$.mp. [mp=title, abstract, heading word, drug trade name, original title, device manufacturer, drug manufacturer, device trade name, keyword, floating subheading word, candidate term word]
6	1 or 2 or 3 or 4 or 5
5	Immediate loading.mp. [mp=title, abstract, heading word, drug trade name, original title, device manufacturer, drug manufacturer, device trade name, keyword, floating subheading word, candidate term word]
4	tooth prosthesis/ or exp "dental prosthesis and implant"/
3	(Immediate interim adj5 (Crown or restoration or prosthesis)).mp. [mp=title, abstract, heading word, drug trade name, original title, device manufacturer, drug manufacturer, device trade name, keyword, floating subheading word, candidate term word]
2	(Immediate temporary adj5 (Crown or restoration or prosthesis)).mp. [mp=title, abstract, heading word, drug trade name, original title, device manufacturer, drug manufacturer, device trade name, keyword, floating subheading word, candidate term word]
1	(Immediate provisional adj5 (Crown or restoration or prosthesis)).mp. [mp=title, abstract, heading word, drug trade name, original title,

	device manufacturer, drug manufacturer, device trade name, keyword, floating subheading word, candidate term word]

Supplementary Table 3. Full data extraction table.

Author & publication year	Gjelvold et al. 2017	Den Hartog et al. 2016	Heydecke et al 2019	Hall et al. 2007	Raes et al. 2018
**Title of study**	Clinical and radiographic outcome following immediate loading and delayed loading of single-tooth implants: Randomized Clinical Trial.	Immediate Loading of Anterior Single- tooth Implants Placed in Healed sites: Five Year Results of a Randomized Clinical Trail.	A prospective multicenter evaluation of immediately functionalized tapered conical connection implants for single restorations in maxillary anterior and premolar sites: 3- year results	Immediately Restored. Single tapered Implants in the anterior Maxilla: Prosthodontic and Aesthetic Outcomes after 1 year.	A long-term prospective cohort study on immediately restored single tooth implants inserted in extraction sockets and healed ridges: CBCT analysis, soft tissue alterations, aesthetic ratings, and patient- reported outcomes
**Journal of study**	Gjelvold, B., Kisch, J., Chrcanovic, B.R, Albrektsson, T. (2017) Clinical and radiographic outcome following immediate loading and delayed loading of single-tooth implants: Randomized clinical trial. Clin Implant Dent Relat Res; 19: 549-558.	Den Hartog, L. Raghoebar, G.M, Stellingsma, K., Vissink, A., Meijer, H.J.A. (2016) Immediate Loading of anterior Single-tooth Implants Placed in Healed Sites: Five-year results of a Randomization Clinical Trial. The International Journal of Prosthodontics; 29(Vol): 584-591.	Heydecke,G., Mirzakhanian, C., Behneke,A., Behneke,N., Fugl,A., Zechner,W., Baer,R.A., Nolken,R., Gottesman, E.(2019). A prospective multicentre evaluation of immediately functionalized tapered conical connection implants for single restorations in maxillary anterior and premolar sites: 3-year results. Clinic Oral investigations;23: 1877-1885	Hall, J.A.G., Payne, A.G.T., Purton, D.G., Torr B., Duncan, W.J, Kumara De Silva, R. (2007) Immediately Restored, Single – Tapered Implants in the Anterior Maxilla: Prosthodontics and Aesthetic outcomes After 1 year. Clinical Implant Dentistry and Related Research 9(Vol): 34-45.	Raes,S. Eghbali, A., Chappuis, V., Raes, F., Bruyn, H.D., Cosyn, J.,(2018) A long-term prospective cohort study on immediately restored single tooth implants inserted in extraction sockets and healed ridges: CBCT analyses, soft tissue alterations, aesthetic ratings, and patient-reported outcomes.Clin Implant Dent Relat Res. 20: 522-530

**Aims and Objective**	To compare the overall treatment outcome between an immediate and delayed loading of single implant	To compare outcome of immediate loading with that of conventional (delayed) loading of single implant	Investigated immediately provisionalized, anodized, conical connection, tapered implants with platform shifting in maxillary anterior and premolar sites. The primary objective is to evaluate the marginal bone loss. Additional secondary outcome measures include implant success and survival, soft-tissue health, esthetics and oral health impact.	To evaluate prosthodontic and aesthetic peri-implant mucosal outcomes of immediately restored, single tapered implants in the anterior maxilla after 1 year	To evaluate immediately restored single implants after 8 years follow-up in terms of buccal bone, soft tissue alterations, aesthetic ratings, and patient-reported outcomes.
**Study design**	Randomized controlled trial with parallel groups	Randomized controlled trial with parallel groups	Prospective cohort study	Randomized controlled trial with parallel groups	Prospective cohort study
**Number of centres**	One	One	Eight	One	One
**Location of study**	Malmo<br>Sweden	Groningen<br>Netherlands	Austria, Germany, Italy, Serbia, and the USA.	New Zealand	Ghent
**Number of participants**	50	62	94	28	23

**Participant allocation/age/ gender**	Parallel design<br>**25 –Immediate Load** Mean age 40.8±13.3(19.0- 66.6)<br>14 Men/ 11Women<br>**25 – Delayed Load** Mean age 40.9± 15.5(18.5-76.7)<br>6 Men/ 19 women	Parallel design<br>**31 –Immediate Load –**mean age 38.4 ± 14.0 (18 -66)<br><br>**31 – Delayed Load-mean** age 40.1 ± 14.4 (18-67)	Prospective Single Cohort<br>99 implants- placed in 94 patients (5 patients received 2 implants each)<br>57.4% female and 42.6% male<br>Mean age 41.1±14.3 years (range 18- 79 years)<br>85.1% non-smokers<br>14.9% smoked previously or throughout the trail.<br>4.2% had history of periodontitis.	Parallel design<br>14 –Conventional restoration group<br>14 – Immediate restoration group<br>No statistical difference in both the groups between age, gender, and smoking status<br>Mean age 43.3 years (range 21-71 years)	Prospective Cohort<br>**IIT** – Immediate Implant treatment – 16 (10 men, 6 women; mean age 45; age range 22-68)<br>**CIT**- Conventional implant treatment – 23<br>(12 men, 11 women; mean age 40; age range 19-75)

**Inclusion criteria**	-At least 18 years age<br>- Single tooth replacement (incisor, canine or premolar) in maxilla<br>- Adjacent natural tooth to be present	At least 18 years age<br>Single tooth replacement (incisor, canine or first premolar) in maxilla<br>Adjacent Natural teeth<br>Adequate oral hygiene (plaque index and bleeding index ≤1)<br>Mesial -distal interdental space ≥6 mm<br>Vertical dimensions allowing a non-occluding temporary crown.	Patient to be ≥18 years of age.<br>In good physical and mental health<br>Committed to complete the full 5-year term including adherence to the scheduled clinical and radiographic analyses and maintenance.<br>The tooth at the implant site had to have been extracted or lost ≥2 months prior to the implantation.<br>Implantation site had to be healthy (free from ongoing active lesions with no tooth remnants, cysts, granulomas, previous tumors or cancer, or undergoing radiation therapy.<br>Full mouth bleeding on probing and full-mouth plaque index had to score ≤25%.<br>Patient had to have favorable and stable occlusal relationship.<br>The implant site had to be adjacent to natural roots.<br>The 1-stage procedure with immediate temporization without full occlusal loading had to be indicated for the patient's condition	Adequate bone volume (length 10-15mm, diameter 2.5-4mm).<br>Lekholm and zarb class I to III bone quality (by radiographic assessment)<br>Healed recipient site<br>Presence of adjacent mesial and distal teeth.	At least 18 years old<br>Minimum 20 teeth present.<br>Good oral hygiene defined as a full mouth plaque score≤25%<br>Presence of single failing tooth or single tooth gap in the anterior maxilla 15-25 with both neighboring teeth present.<br>Ideal soft tissue levels and contours on facial aspect, implying no visible disparity between the latter and the contra-lateral tooth and adjacent teeth.<br>Appropriate bone volume as assessed by standard radiographs or CBCT scans.<br>Signed consent.

**Exclusion criteria**	-general contraindications for oral surgery<br>-patients with inadequate oral hygiene<br>- need for bone grafting or ridge augmentation at the implant site<br>- exposure of more than 1mm of implant during surgery<br>- insertion torque below 30Ncm was excluded for Immediate implant group.	Presence of active periodontal disease (pocket depth of ≥ 4mm, bleeding on probing)<br>Presence of periapical lesions of any other abnormalities<br>Smoking<br>A history of radiotherapy of head and neck	Acute untreated periodontitis; health conditions preventing surgical treatment.<br>Any disorders in the planned implant area. E.g., previous tumors, chronic bone disease (rheumatoid disease)<br>Infections in tissues adjacent to the planned implant sites<br>previous oromaxillofacial radiotherapy<br>Use of interfering medication (steroids or bisphosphonates)<br>Alcohol or drug abuse<br>Heavy smoking (> 10 cigarettes /day)<br>Uncontrolled diabetes<br>Severe bruxism<br>Destructive habits<br>Pregnancy or lactation<br>**Secondary exclusion**: At the time of surgery insufficient bone volume at the implant site to place a ≥3.5 mm diameter, 8 mm length implant.<br>The need for major bone augmentation at the implant site<br>Insertion torque ≤35 or≥45 Ncm.	Heavy smoking<br>Severe bruxism or clenching habits<br>Physical or mental disabilities that would interfere with maintenance of implants.<br>Previous history of failed implants<br>*Untreated periodontitis*<br>*After Implant placement if no primary stability or if bone required grafting or ridge augmentation procedures prior to implant placement were excluded from the study.*	Pregnancy at the time of inclusion<br>Uncontrolled diabetes mellitus<br>Smoking<br>Non-treated periodontal disease and/or caries<br>Immediate implant treatment (**IIT)**- incomplete buccal bone wall or thin gingival biotype<br>Conventional implant treatment (**CIT**)- failing tooth has been extracted less than 3 months, sites that are treated by guided bone regeneration.<br>Primary implant stability less than 25 NCM
**ASA score**	Not Given	ASA Score ≤ 111	Not known	Not known	Not known
**Ethical approval**	Obtained	Obtained	obtained	obtained	Obtained
**Consent**	Obtained	Obtained	Obtained	obtained	Obtained

**Clinical examination and investigations**	Examination and radiographs were taken before randomization	Examination and radiographs were taken before randomization	Not known	Periapical, panoramic radiographs and cross -sectional tomographs were used to evaluate bone quantity and quality to determine implant length. Prosthodontic consultation was carried out	After clinical examinations patients were allocated to IIT or CIT group. Panaromic radiographs, peri-apical radiographs, CBCT were taken prior to allocation. Bone quality and quantity were assessed
**Method of Randomization**	Closed randomization with sealed envelops	Locked computer software was used for randomization. Randomization by minimization was performed. Surgeon was informed of the allocation on the day of the surgery.	Not applicable	Closed randomization using sealed envelops	Not applicable
**Factors considered for minimization**	Not done	Age ≤ 30,> 30≤ 60 or >60 Location of implant (incisor, canine or premolar) Indication of peri-implant bone augmentation	Not applicable	Not done	Not applicable

**Person carrying out the treatment**	**Surgeon** -Surgeon 1 / blinded.<br>**Prosthetic procedures**: Dentist 1<br>**Outcome assessment**: Dentist 1<br>**Technician**: One<br>**Statistical examination**: Examiner 1	**Surgeon** -Surgeon 1 / blinded until surgery.<br>**Prosthetic procedures**: Prosthodontist 1 and 2<br>**Outcome assessment**: Examiner blinded.<br>**Photographer**: One examiner blinded<br>**Technician**: One	**Surgeon:** 8 participating centers**,** all surgeons were experienced**.**	**Surgeon** - One Experienced Surgeon (R.K) placed 15 Implants – 9 Conventional restoration group.<br>6 Immediate restoration group.<br>He supervised an inexperienced graduate student in periodontology who placed 13 implants- 5 conventional restoration group.<br>8- Immediate restoration group.<br>**Prosthetic procedures**: Dentist 1<br>**Technician**: One	**Surgeon** -periodontist 1<br>**Prosthetic procedures**: Prosthodontist 1<br>**Outcome assessment**: Dentist 1<br>**Technician**: One
**Type of Implant placed**	Tapered Internal Implants (BioHorizons)	Nobel Replace Tapered Groovy Implant (Nobel Biocare)	Anodized, tapered implants with an internal hexagonal interlocking conical connection and built-in platform shifting (NobelReplace Conical Connection, Nobel Biocare)	Southern implants-tapered implants (2.5-4 mm)<br>Roughened surface<br>Sa of 1.43µm (giving a developed surface area of 50%<br>Threads were 1mm part with a pitch of 10 degree.<br>Made of grade 4 commercially pure titanium	Astra tech implant system, Osseospeed.
**Site of Implants**	Incisor, Canine or premolar in Maxilla	Incisor, Canine or first premolar in Maxilla	Incisors, canine, first premolar and Second premolar.	Incisor, Canine, first premolar or second premolar in Maxilla	Incisor, Canine, first premolar or second premolar in Maxilla
**Site of Implant**	Healed Bone (4 or more months after extraction)	Healed Bone (3 or more months after extraction)	Healed bone (≥2 months after extraction)	Healed bone	Healed bone (more than 3 months after extraction)

**Prophylactic antibiotics**	Yes<br>Phenoxymethylpenicillin 500mg 8/8 hours<br>1 hour before surgery	Not mentioned	Not mentioned	Not Mentioned	Not known
**Surgical procedure**	Standardized Surgical Procedure<br>LA<br>Mid crestal with vertical releasing incision	Implants were placed and restored according to protocol reported in Den Hartog et al 2009.	Flaps were lifted in most cases. 72.7% not using releasing incision.<br>23.2% using releasing incision.<br>4% were inserted flapless procedure.<br>**Soft tissue grafting was performed in 13 implant sites (13.1)**<br>16.2% implants required bone grafting: 8 with autologous bone and 8 with xenograft material.<br>5.1% implant sites had undergone bone grafting [illegible]	Intra-sulcular incision - including interdental papillae of adjacent teeth through mid-crest.<br>Vertical relieving incision made from the distal papillae.<br>Labial mucoperiosteal flap was elevated to expose bone.	**CIT**- minimal mucoperiosteal flap extending midfacial aspect of both neighboring teeth in 5/23 patients.<br>Full thickness mucoperiosteal flap in 18/23 patients<br>Mid crestal incision
**Surgical guide stent**	Not known	A surgical template was used to install the implants at a depth of 3mm apical to the buccal and cervical aspect of crown	Not known	Used to position each implant	Not used

**Implant Placement**	Following manufacturer recommendations	Implants placed with torque controller to an insertion torque of 45Ncm and manual torque controller was used	The insertion torque had to be between 35 and 45 Ncm as measured using a manual torque wrench. Implant stability at implant insertion was tested by tapping and rocking the implant with a hand instrument.	Osteotomy was performed with round bur.<br>2mm pilot drill and tapered final drill.<br>Implants placed at a depth of 3-4mm from the expected labial mucosa emergence, depending on the tissue biotype.<br>In immediate group implant head bone mill was used to ensure unhindered access for prosthodontic components	Implants placed in correct 3dimensional position with 25Ncm torque.<br>In the mesiodistal dimension: a minimum of 2mm between the implant shoulder and neighboring tooth<br>In the orofacial dimension – implant shoulder was positioned palatal to the point of emergence at the adjacent teeth.<br>Apico-coronal direction: 1-2 mm apical to the cemento enamel junction of adjacent tooth
**Inspection for Buccal bone fenestrations dehiscence Exposure of Implant**	< 1 mm were covered with autogenous bone chips collected during implant bed preparation	Implant dehiscence and fenestrations were covered with autogenous bone chips and an organic bovine bone	Bone quality and quantity were assessed at the time of implant placement according to the Lekholm and Zarb classification. The needs for bone and soft-tissue grafting at the time of implant placement was determined on a case-by-case basis. The grafting methods used were left to the surgeon's discretion.	Buccal fenestrations and dehiscence were covered with autogenous bone grafts recovered from osteotomy and immediate surgical site.<br>**Conventional group**: 4 implants had buccal fenestrations (3-7 threads exposed)<br>1 implant had dehiscence.<br>**Immediate restoration group:** 5 implants had buccal fenestrations and 2 had buccal dehiscence.	All patients in the CIT group had sufficient bone no grafting was needed
**Membrane used**	No	Yes	Not known	No	No
**Post-operative antibiotics**	7 days	Not Mentioned	Not known	Not known	Amoxicillin 500mg three times per day -5 days

**Post-operative care**	-0.2% chlorhexidine twice daily for 14 days<br>-analgesics if needed Paracetamol 500mg 6/6 hours	Not Mentioned	Not known	Mouth rinse with 0.2% chlorhexidine for 2 weeks and begin soft brushing the provisional crowns or surgical site once the pain subsides	Oral hygiene reinforced and paracetamol 100mg. Chlorhexidine mouth wash 0.2% - 2 times a day for 1 week
**Type of immediate temporary crown**	Screw Retained – Titanium temporary abutment with composite crown	Screw retained temporary crown	A cement- or screw-retained provisional<br>86 implants- temporary abutment with temporary crown were placed immediately.<br>13- final abutments with temporary crowns were placed immediately	Screw retained provisional crown.<br>An innovative technique was used: The acrylic denture tooth facing, that has been used previously in the diagnostic wax-up was bonded using composite to a silanated hexed titanium cylinder. The denture tooth facing was positioned on the cast made from the surgical impression, using a silicone index made on the diagnostic wax-up.	Titanium direct abutment and acrylic crown<br>Cement retained provisional crown with temporary cement (temp bond)
**Timing of Immediate temporary crown**	Immediate	Within 24 hours of implant placement	Temporization and functionally loaded within 24 hours following surgery	Within 4 hours of Implant surgery	Immediate

**Occlusion of immediate temporary crown**	Light centric contact Free from eccentric contact s with opposing teeth	Crown was free from centric contacts with antagonist teeth	Functionally loading	Free of contacts in maximum intercuspation and excursions, using a 200µm articulating paper. All provisional crowns had proximal contacts except where diastema was required for esthetic reasons	Clear of all contacts in centric occlusion and eccentric movements.
**Tightening of temporary crown**	15 Ncm	Not mentioned	56 immediate – cemented.<br>43- screw retained	Not mentioned	Not applicable
**Post-operative care for Immediate temporary crown (loading)**	Instructions to avoid exerting force on temporary crown	Soft Diet and avoid exerting force on temporary crown	Not known	Refrain from chewing on the implant crown for 12 weeks	Avoidance of the surgical site<br>Oral hygiene
**Delayed provisional crown and healing period.**	4 months	3 months of healing	Not applicable	26 weeks of healing. An implant bone mill was used to provide access to prosthodontic components	Not applicable
**Type of delayed temporary crown**	Screw Retained – Titanium temporary abutment with composite crown	Screw retained temporary crown	Not applicable	Screw retained provisional crown.<br>An innovative technique was used: The acrylic denture tooth facing, that has been used previously in the diagnostic wax-up was bonded using composite to a silanated hexed titanium cylinder. The denture tooth facing was positioned on the cast made from the surgical impression, using a silicone index made on the diagnostic wax-up.	Not applicable

**Soft tissue conditioning with temporary crown**	Crown shape and emergence profile modified in Delayed loading group	No soft tissue conditioning carried out	Not known	Technician created an ideal definitive emergence profile for the provisional crown and replicated this with the definitive crown to support the healing peri-crown mucosa	No soft tissue conditioning
**Timing of definitive crown in immediate loading group**	2 months from implant installation	6 months from Implant installation	Within 6 months after implant placement	8 weeks pf provisionalization	10 weeks following implant surgery
**Timing of definitive crown in delayed loading group**	After 8 months of Implant placement	6 months from Implant installation	Not applicable	8 weeks pf provisionalization	Not applicable
**Impression technique for definitive crown**	Implant level impression with customized impression coping to copy the emergence profile.	Not Mentioned	Not known	Implant level impression using vinyl polysiloxane with impression coping	If the direct abutment provided adequate retention, then Impression of the direct abutment carried out if more retention was necessary then then it was replaced with titanium TiDesign abutment and Polyether impression made.
**Definitive crown**	Customized Zirconia abutment with titanium base, cemented or screw retained	Customized Zirconia abutment cemented, or screw retained	Cement or screw retained NobelProcera crown with a titanium or zirconia abutment (decision regarding type of abutment and prosthetic retention method was left to the surgeon's discretion to ensure they met the individual patient's needs.	Screw retained metal ceramic crown on hexed old cylinders	Titanium abutment and full ceramic crown<br>Cement retained crown (cemented with Glass Ionomer cement)

**Follow up by hygienist**	For 6 months from implant placement	Not mentioned	Not mentioned	Not mentioned	Not mentioned
**Follow up**	3,6, 12 after definitive crown placement	1 year and 5 year after definitive crown placement	6 months, 1-2-3 year follow up	12 weeks after implant placement for peri-implant measurement in Immediate group<br>36 weeks after implant placement for peri-implant measurements in delayed group.<br>12 months recall	After 1 year and 8 years
**Esthetic outcome measures**	Primary (PES, WES, and Papilla Index)	Secondary (PES, WES, and Papilla Index)	Secondary outcome) Papilla Index and PES)	Primary (Papilla Index)	Primary (PES and WES)
**Base line For aesthetic outcome**	Day of definitive crown placement	Final crown placement	32.8% had acceptable PI scores of 2 or 3- at placement.<br>Mean PES of definitive prosthesis at placement was 8.48 ±1.91.	4 weeks after definitive crown placement for aesthetic and papilla index	Not taken

	Photographs taken using NikonD7000 by technique described by Furhauser et al 2005.	Full screen analysis of photographs was performed using Adobe photoshop. Photographic procedure as described by Meijndert et al.	All soft- tissue parameters were assessed by an independent evaluator (Vienna Medical University, Austria)		Digital slides and computer software (Gingival status 2009). The angle of the camera was set so patient is positioned in same position at each assessment.
**Photographs for PES (Pink Esthetic score and WES (White esthetic score)**	taken at baseline and follow up appointments for PES.<br><br>Photographs taken at final follow up for WES.	taken at baseline, 1 year and 5 year follow up for both PES and WES.	*There is no mention of photographic technique or procedure.*	Not an outcome measure	Frontal and occlusal color digital slides taken at 4,12,26 weeks, 1 and 8 years
**Cast for papilla index. Circumferential mucosal line**	Yes, Cast were made for temporary and permanent restoration (3,6,12 months)	Not mentioned	No mention of the technique	No Cast Circumferential mucosal line was marked on the definitive crowns at the gingival margins to facilitate accurate mucosal measurements	Not an outcome measure

**PES at 1st review in Immediate loading group (mean ± SD (min-max))**	10.36 ±2.46 (3-14)<br>At 12 months	7.1± 1.5(3-10)<br>At 12 months	Mean PES at 1 year follow up- 9.69 ±2.04.	Not an outcome measure	*Mean PES at 1 year review 10.35 (SD 1.58, range 7-13) for 23 patients (According to 1 st paper)*<br>*Recent paper shows Mean PES 9.70(1.72) for 20 patients*
**Improvement in PES in Immediate loading group**	Statistically significant improvement between Initial evaluation to 1 year follow up in PES. (P = .001)	Not Known	P <0.0001 compared with definitive prosthesis placement.	Not an outcome measure	*P = 0.763 between PES at 1 year and 8 years*
**PES at 2, 3,5 0r 8 years in Immediate loading group (mean ± SD (min-max))**	Not known	7.2 ±1.5(3-10) at 5 years	At 2 years – 10.04 ±1.98<br>At 3 years - 9.87± 2.19	Not an outcome measure	At 8 years 9.22(2.31)<br>For 18 patients

**PES at 1st review in delayed loading group (mean ± SD (min-max))**	10.67 ± 2.32(5-14)<br>At 12 months	6.5 ± 1.6 (4-9)<br>At 12 months	Not a Cohort	Not an outcome measure	Not a Cohort
**Improvement in PES in Delayed loading group**	Statistically significant improvement between Initial evaluation to 1 year follow up in PES.<br>(P = .002)	Not known	Not a Cohort	Not an outcome measure	Not a Cohort
**PES at 5 years in delayed loading group (mean ± SD (min-max))**	Not Known	6.8 ±1.3(5-9)	Not a Cohort	Not an outcome measure	Not a Cohort
**WES after 12 months in Immediate loading group (mean ± SD (min-max))**	7.76 ± 1.30(5-10)	7.8 ±1.5(4-10)	Not an outcome measure	Not an outcome measure	7.00(SD 2.37) (2-10)
**WES at 5 years in Immediate loading group (mean ± SD (min-max))**	Not known	7.9± 1.2 (5-9)	Not an outcome measure	Not an outcome measure	Not given
**Improvement in WES in Immediate loading group**	Statistically significant improvement between Initial evaluation to 1 year follow up in WES.<br>(P = .008)	Not known	Not an outcome measure	Not an outcome measure	Data for 8-year WES is not available

**WES after 12 months in Delayed loading group (mean ± SD (min-max))**	7.87 ±1.39(5-10)	7.6±1.6(4-10)	Not an outcome measure	Not an outcome measure	Not applicable
**WES at 5 years in Delayed loading group (mean ± SD (min-max))**	Not known	7.7± 1.2(5-10)	Not an outcome measure	Not an outcome measure	Not applicable
**Improvement in WES in Delayed loading group**	Statistically significant improvement between Initial evaluation to 1 year follow up in WES. (P = .001)	Not Known	Not an outcome measure	Not an outcome measure	Not applicable
**P Value for PES at 1st review between Immediate load and delayed load**	P= 0.700<br>At 12 months	P>0.05<br>At 12 months	Not applicable	Not an outcome measure	Not applicable
**P Value for PES at 5 years between Immediate load and delayed load**	Not known	P>0.05	Not applicable	Not an outcome measure	Not applicable

**P Value for WES at 12 months between Immediate load and delayed load**	P= 0.724	P>0.05	Not an outcome measure	Not an outcome measure	Not applicable
**P Value for WES at 5 years between Immediate load and delayed load**	Not known	P>0.05	Not an outcome measure	Not an outcome measure	Not applicable

**Mean Distance until complete papilla fill (According to papilla Index) for Immediate load group.**	0.77±0.71 mm	Not Known	Not mentioned	Not known	Not an outcome measure
**Percentage of patients with complete papilla fill mesial and distal sides in Immediate load after 12 months or more.**	At 12 months - 28%	At 12 months - 43.3 %	Acceptable PI scores (2 or 3) in 87.5% at 6 months<br>90.6% at 1 year<br>92.8% at 2 years<br>88.6% at 3 years	Not known	Not an outcome measure
**Percentage of patients with complete papilla fill mesial and distal sides in Immediate load at 5 years**	Not known	36.5%	Not yet been 5 years	Not known	Not an outcome measure

**Mean Distance until complete papilla fill (According to papilla Index) for Delayed load group.**<br><br>**Percentage of patients with complete papilla fill mesial and distal sides in delayed load after 12 months**	0.60±0.74 mm<br><br>46%	Not Known<br><br>33.9%	Not a cohort<br><br>Not a Cohort	Not known<br><br>Not known	Not an outcome measure<br><br>Not an outcome measure
**Percentage of patients with complete papilla fill mesial and distal sides in delayed load at 5 years**	Not known	25.9%	Not a cohort	Not known	Not an outcome measure

**Number of participants with complete mesial papilla fill at placement and at 1 year.**	Not mentioned	Not mentioned	Not mentioned	Combined number of patients: 4<br>Combined number at 1 year: 7	Not an outcome measure
**Number of participants with complete Distal papilla fill at placement and at 1 year**	Not mentioned	Not mentioned	Not mentioned	Combined number at Placement: 3<br>Combined at 1 year: 5	Not an outcome measure
**P Value of Papilla index, mesial for definitive crown/ 12 months between Immediate load and delayed load**	P= 0.533	Not Known	Not mentioned	No statistical difference	Not an outcome measure
**P Value of Papilla index, distal for definitive crown/ 12 months between Immediate load and delayed load**	P= 0.366	Not Known	Not mentioned	No statistical difference	Not an outcome measure

**P Value of Papilla index, Complete papilla fill at 12 months between Immediate load and delayed load**	P= 0.244	No statistical difference	P <0.0001 compared with placement.<br>P >.25 between follow up visits	No statistical difference	Not an outcome measure
**Loss to follow-up/Excluded**	No dropouts<br>One Implant lost in delayed loading group 3 months after surgery(smoking)<br>Two patients missed 6 months follow up.<br>Immediate group = 25 at 1 year<br>Delayed Group = 24 at 1 year	One implant lost in Immediate group after temporary crown (mobility)<br>3 lost to follow up at 5 years in both groups.<br>Clinical data not available for 1 in immediate group at 5 years<br>Immediate group = 26 at 5 years<br>Delayed Group = 27 at 5 years	101 were initially enrolled – 5did not meet inc/ex criteria.<br>One was removed at the time of surgery due to complications.<br>One patient was removed after 1 year left with 94 patients.<br>3 implants/patients missed. Prosthesis delivery appointment (were withdrawn prior to delivery)<br>92implants were looked at 6 months.<br>91 implants at 1 year<br>84 at 2 year<br>88 at 3 years	2 participants from conventional group failed to return at 1 year follow up.<br>1 participant in immediate group failed to return 1 year follow (but was available over phone)<br>1 implant failed in immediate group and was removed at 10 weeks post-surgery.	5 out of 23 did not come for a review at 8 years.<br>Study mentions combined reasons for loss of follow up of both IIT and CIT patient<br>1 – implant failure<br>2- patient not willing to return.<br>6-patients unreachable<br>1- Patients deceased

**Conclusion**	The papilla index score between both the groups was statistically significant. This could be due to the difference in the timing of placement. The delayed group had the benefit of soft tissue conditioning compared to Immediate group.<br>The recall period was set to 12 months, if the follow up was longer then the results would show additional papilla formation.<br>PES and WES show statistically significant improvement from baseline. However, there is not much difference in between both at 12 months follow up.	There is no difference in esthetic outcome between immediate and delayed loading group using PES/WES /Papilla Index score<br>The reason claimed for no difference is implants placed in healed sites, the authors suggest that any positive effects of immediate loading on peri-implant mucosa is subsided due to healed sites.<br>No comparisons between baseline esthetics to final esthetics were mentioned to show the effect of provisional crown.	The study had a previous paper published with 1 year follow up results. The scores given for 1 year do not match the ones in the 3-year report.<br>This study was carried out in different centers by different surgeons. There is variable skill set and different technicians.<br>The surgical procedure and prosthetic decision were left to the induvial practitioners.<br>Some implants received soft tissue grafting. This could have an effect over the esthetic outcome. Especially in patients with thin gingival biotype. The study shows that PES and PI scores have improved gradually and remained stable.	The study concluded that no statistical difference in papilla index between the groups or within the group.<br>The study presented combined results for immediate and conventional group. Papilla index (mesial and distal) for both groups combined remained unchanged in 28.5% of sites and improved in 63% of sites.<br>No statistical values for esthetic outcome were given. The study had one experienced surgeon and another trainee. 5 Implants (3 conventional and 2 immediate) were positioned palatal causing to be restored with subgingivally receding buccal profile crown. However, in the aesthetic outcome measures this was not mentioned.<br>The overall conclusion was that esthetic outcome did not differ between both the groups over 1-year period. The recall perios is short along with small number of participants could contribute to a statistical error	The outcome of this prospective cohort study has been reported in another case series report. The mean PES at 1 year reported was 10.35 for 23 patients. However, in the 8-year prospective study the mean PES at 1 year reported for 20 patients is 9.70. The study reports that there is no statistically significant difference between 1 year and 8 years ($p \geq 0.470$). The study recruited high risk patients with a thin -scalloped gingival biotype and thin bone wall into CIT group owing to the low mean PES value at 8 years

Supplementary 4. Table of excluded studies

Number	Study	Reason
1.	Donos, N., Horvath,A., Calciolari,E., Mardas, N. (2018). Immediate provisionalization of bone level implants with a hydrophilic surface. A five-year follow up of a randomized controlled clinical trial. Clin Oral Impl Res: 30;139-149.	This Randomized controlled study compared Immediate provisionalization with a non-occluding temporary crown with delayed direct definitive crown. Comparator is different.
2.	Degidi,M., Nardi,D., Piatteli,A.(2009) Immediate Versus one-stage restoration of small-diameter implants for a single missing maxillary lateral incisor- A 3- year Randomized Clinical trial. J periodontal 2009; 80: 1393-1398.	This Randomized controlled study compared Immediate provisionalization with a non-occluding temporary crown with delayed direct definitive crown. Comparator is different.
3.	Heinemann, F., Grufferty, B., Papavasiliou, G., Dominiak, M., Garcia, J.J., Trullenque-Eriksson, A., Esposito,M. (2016). Immediate occluding definitive partial fixed prosthesis versus non-occluding provisional restorations- 4- month post-loading results from a pragmatic multicentre randomised controlled trail. Eur J Oral Implantol; 9(1):47-56.	This Randomized controlled study compared Immediate provisionalization with a non-occluding temporary crown with definitive occluding partial fixed prosthesis within 1 week. Comparator is different
4.	Yildiz,P., Zortuk,M., Kilic,E., Dincel,M., Albayrak,H.,(2018) Esthetic outcomes after Immediate and late implant loading for a single missing tooth in the anterior maxilla. Niger J Clin Pract; 21:1164-70.	This Randomized controlled study compared Immediate provisionalization with a non-occluding temporary crown with delayed direct definitive crown. Comparator is different.
5.	Belser,I.C., Grutter,L., Vailati,F., Bornstein.M.M., Weber H.P., Buser,D.(2009).Outcome evaluation of early placed maxillary anterior single-tooth implants using objective esthetic criteris: A cross-sectional, Retrospective study in 45 patients with a 2-to 4- year follow- up using pink and white esthetic scores. J Periodontol 2009; 80:140-151.	This case series study looked at early implant placement with provisional restoration at 1-3 moths. Does not meet the inclusion criteria.
6.	Cooper,L.F., Raes,F., Reside,G.J., Garriga, J.S., Tarrida, G.L., Wiltfang,J., Kern,M., Bruyn, HD.(2010). Comparison of radiographic and clinical outcomes following immediate provisionalization of Single-tooth dental implants placed in healed alveolar ridges and extraction sockets. Int J Oral Maxillofac Implants; 25:1222-1232.	This Randomized control trail looked at immediate provisional crown placed in healed sites to provisional placed in extraction sites. Comparator is different
7.	Esposito,M., Barausse,C., Pistilli, R., Grandi,G., Tuco,L., Felice,P.(2015). Immediate loading of post- extractive versus delayed placed single implants in the anterior maxilla: outcome of a pragmatic multicentre randomised controlled trail 1-year after loading. Eur J Oral Implantol;8(4):347-358.	This Randomized control trail looked at immediate provisional crown placed in healed sites to provisional placed in extraction sites. Comparator is different
8.	Kolinski,M., Hess, P., Leziy,S. Friberg,B., Belluci,G., Trisciuoglio,D., Wagner.W., Moerhel, M., Pozzi, A., Wiltfang,J., Behrens, E., Zechner, W., Vasak, C., Weigl, P.,(2018) Immedaite provisionalization in the esthetic zone: 1- year interim results from a prospective single-cohort	This prospective cohort study looked at immediate provisional restoration both in healed and extractions. However, the study presented data from both maxillary and mandibular teeth together.

	multicenter study evaluating 3.0-mm-diameter tapered implants. Clinial oral investigations; 22: 2299-2308.	Mixed data
9.	Payer, M., Arnetzl, V., Kirmeier,R., Koller,M., Arnetzl,G., Jakse,N.(2013). Immediate provisional restoration of single-piece zirconia implants: a prospective case series- results after 24 months of clinical function. Clin.oral. Impl.Res;24: 569-575.	This is a case series reporting both on maxillary and mandibular teeth.<br>Case series and mixed information
10.	Siebers, D., Gehrke,P., Schliephake,H.(2010). Immediate versus delayed fuction of dental implants: a 1-to 7-year follow-up study of 222 implants. Int J Oral Maxillofac Implants; 25:1195-1202.	This prospective cohort study did not report on the aesthetic outcome.
11.	Fu, P.S., Wu,J.C., Huang, T., Chen, Huang ,J.W, Hung, C.C.,(2012)Optimizing anterior esthetics of a single-tooth implant through socket augmentation and immediate provisionalization: A case report with 7-year follow -up. Kaohsiung Journal of medical sciences ;28: 559-563.	Case report
12.	Rieder, D., Eggert, J., Krafft, T., Weber H-P., Wichmann, M.G., Heckmann, S.M. (2016), Impact of placement and restoration timing on single-implant esthetic outcome- a randomized clinical trial. Clin. Oral Impl.Res. 27: e80-e86.	In this Randomized control trail the implants were placed in sites of 4-6 weeks of healing (early placement) as opposed to in healed sites(conventional placement)<br>Does not meet the inclusion criteria.
13.	Donati,M., Scala, V., Billi,M.,Di Dino, B., Torrisi,P., Berglundh, T.(2008). Immediate functional loading of implants in single-tooth replacement: a prospective clinical multicenter study. Clin.Oral Impl.Res.19: 740-748	In this randomized control trail the implants were placed in15-25 and 35-45 position. No esthetic outcomes were reported.
